# Optimization of Submodularity and BBO-Based Routing Protocol for Wireless Sensor Deployment

**DOI:** 10.3390/s20051286

**Published:** 2020-02-27

**Authors:** Yaoli Wang, Yujun Duan, Wenxia Di, Qing Chang, Lipo Wang

**Affiliations:** 1 College of Information and Computer, Taiyuan University of Technology, Jinzhong 030600, China; duanyujun0248@link.tyut.edu.cn (Y.D.); changqing@tyut.edu.cn (Q.C.); 2 Foreign Languages Department, Taiyuan Normal University, Jinzhong 030600, China; wendy_cn@263.net; 3 School of Electrical and Electronic Engineering, Nanyang Technological University, Singapore 639798, Singapore; elpwang@ntu.edu.sg

**Keywords:** wireless sensor deployment, submodularity, ant colony algorithm, routing protocol, biogeography-based optimization

## Abstract

Wireless sensors are limited by node costs, communication efficiency, and energy consumption when wireless sensors are deployed on a large scale. The use of submodular optimization can reduce the deployment cost. This paper proposes a sensor deployment method based on the Improved Heuristic Ant Colony Algorithm-Chaos Optimization of Padded Sensor Placements at Informative and cost-Effective Locations (IHACA-COpSPIEL) algorithm and a routing protocol based on an improved Biogeography-Based Optimization (BBO) algorithm. First, a mathematical model with submodularity is established. Second, the IHACA is combined with pSPIEL-based on chaos optimization to determine the shortest path. Finally, the selected sensors are used in the biogeography of the improved BBO routing protocols to transmit data. The experimental results show that the IHACA-COpSPIEL algorithm can go beyond the local optimal solutions, and the communication cost of IHACA-COpSPIEL is 38.42%, 24.19% and 8.31%, respectively, lower than that of the greedy algorithm, the pSPIEL algorithm and the IHACA algorithm. It uses fewer sensors and has a longer life cycle. Compared with the LEACH protocol, the routing protocol based on the improved BBO extends the life cycle by 30.74% and has lower energy consumption.

## 1. Introduction

Wireless sensors are widely deployed on a large scale in commercial fields [1,2], but are limited by node costs, communication efficiency between nodes, and energy consumption [3,4,5], e.g., in forest and grassland fire risk monitoring and early warning. The problem of wireless sensor deployment is considered as deploying a certain number of nodes to meet monitoring needs, that is, finding the number and location of deployed nodes. The goal of solving this problem is to find as few sensors as possible to meet the monitoring requirements and reduce the communication cost. It is transformed into an optimal sensor node solution set, which is an NP-hard problem. The sensor deployment problem has diminishing returns, e.g., submodularity [6,7,8]. Initially, when a small number of sensors are deployed, each new sensor will significantly improve its deployment utility. As more sensors are placed, the improvement in utility from adding new sensors diminishes. Krause [9] showed that for problems with submodularity, at least the (1−1/e) approximation of the optimal solution can be obtained using the greedy algorithm.

Many methods have been proposed for sensor deployment. In [10], Huang et al. assumed that the node’s perception ability is a circular area. That is, targets within the circular area are fully perceived, and targets outside the circular area will not be perceived. In [11], Guestrin et al. proposed the use of mutual information-based optimization criteria so that the set of deployed nodes contains information about unselected points, and the monitoring is quite accurate. In [12], Cheng et al. proposed a Markov random field model to describe the data correlation between sensor nodes. In [9], Krause deployed sensors with a greedy algorithm to maximize the amount of information, but neglecting the influence of the communication distance between nodes. In [13], Krause et al. improved the greedy algorithm and proposed the Padded Sensor Placements at Informative and cost-Effective Locations (pSPIEL) algorithm to solve the problem of sensor deployment optimization under the constraint of communication distance; however, a large number of sensors needed to be deployed. In [14], Mariohat et al. established a Gaussian model, improved the greedy algorithm under the constraint of fixed costs, and proposed the SUPSUB method to minimize the submodular set function, while neglecting the influence of the communication distance between nodes on the deployment. The sensor placement problem considering communication distance is a constrained optimization problem. The bi-projection neural network proposed by Xia et al. [15] can effectively solve large-scale constrained optimization problems, and has good stability and faster convergence [16]. Liu et al. [17] proposed a ML-OAXSMT-PSO construction algorithm, which can significantly reduce the total cost.

During communication transmission, wireless sensors have limited energy, but effective clustering nodes can better save energy and extend the life cycle of the entire network. There are various energy-saving methods. Guo et al. [18] proposed the FTAOA algorithm to minimize task completion time to save node energy. Cheng et al. [12] proposed the NSA algorithm to reasonably deploy nodes and significantly improve network lifetime. Liu et al. [19] proposed the KPNS algorithm to appropriately select more active nodes for monitoring, so that the energy is fully utilized. Effective node clustering can greatly save energy and extend the life cycle of the entire network. The LEACH protocol balances the energy of each sensor in the entire network by randomly selecting cluster heads [20]. However, it has the problem of uneven number and distribution of cluster heads [21], for not having considered the transmission distance. This causes either the nodes far away from the base station to be selected as the cluster head or the nodes far away from the cluster head to die prematurely [22]. In [23], Simon proposed the biogeography-based optimization algorithm with advantages of simple operation, few parameters, and high search accuracy [24]. In [25], Pal and others used the Biogeography-Based Optimization (BBO) algorithms to select cluster heads and cluster nodes, and obtained good energy efficiency. However, the authors only took the distance between cluster heads and the distance between nodes in the cluster into consideration, while neglecting the energy consumed by data transmission between nodes.

Deploying wireless sensors is limited by cost and power consumption [26]. Therefore, the following two issues need to be considered during deployment: one is to achieve efficient data collection; the other is to use as few sensors as possible and minimize the communication distance between sensors to reduce the total energy consumption. Because of the existing problem of deploying fewer sensors in terms of the distance between nodes, and the deficiency of some popular algorithms in the field of sensor deployment, we shall proceed as follows in this paper. The mutual information is used to describe the correlation between the observed and the unobserved points. The communication distance is described by the connection of the graph and the Improved Heuristic Ant Colony Algorithm-Chaos Optimization of Padded Sensor Placements at Informative and cost-Effective Locations (IHACA-COpSPIEL) algorithm is used to choose the optimized submodular model. By considering the distance between clusters, the distance between nodes in the cluster, and the energy consumption of data transmission by the nodes, we obtain an optimized routing protocol in which the BBO algorithm is used to transmit data with an improved cost performance.

The structure of this article is as follows. In Section 2, wireless sensor deployment optimization is introduced. The routing protocol of the wireless sensor network based on the BBO algorithm is presented in Section 3. In Section 4, we introduce the experimental verification, discuss the deployment effects using different algorithms, and analyze the performance of the protocol for the BBO algorithm. Finally, this article is summarized in Section 5.

## 2. Wireless Sensor Deployment Optimization

### 2.1. Wireless Sensor Deployment

#### 2.1.1. Problem Description

Given a certain area of interest, V(|V|=N) is the set of monitored locations in the area. The goal of node deployment is to select a subset A⊂V(|A|=K<N), and the base station can efficiently estimate the value of any element in the set V\A(|V\A|=N−K) based on the observed values of the subset.

Mutual information is a way to describe the correlation between two sets of events. This paper uses mutual information to describe the correlation between set A with deployed sensors and set V\A without deployed sensors. Suppose set $V=[V_{1},V_{2},\ldots ,V_{N}]$ represents *N* positions, and $X_{V}=\left[X_{1},X_{2},\ldots ,X_{N}\right]$ describes the random variables of the observation results of these positions. For any subset A⊂V, $X_{A}$ is used to represent the set of random variables associated with the location subset *A*. The objective function is to maximize the amount of information containing the unselected location set through the selected deployment set *A*.

(1) $$\max_{A\subseteq V}F\left(A\right)=\max_{A\subseteq V}\left(H\left(X_{V\backslash A}\right)-H\left(X_{V\backslash A}|X_{A}\right)\right).\;$$

In Equation (1), $H(X_{V\backslash A})$ represents the entropy of the random variable $X_{V/A}$, and $H(X_{V\backslash A}|X_{A})$ represents the random variable $X_{V/A}$ relative to the conditional entropy of $X_{A}$.

#### 2.1.2. Objective Function of Sensor Deployment Considering Communication Cost

When deploying sensors, we must consider not only the number of sensors, but also the energy consumption during wireless sensor network transmission, because energy consumption is related to the distance between sensors. However, we cannot determine which sensors will communicate to accurately reduce the communication distance between them. Therefore, the communication distance between all the sensors is selected to be reduced so that the communication distance between subsequent sensors is reduced. Assume that there are *N* optional points. Node *i* and node *j* are selected to deploy sensors. The communication cost between the two nodes is defined as follows.

(2) $$d_{i,j}=\sqrt{{\left(x_{j}-x_{i}\right)}^{2}+{\left(y_{j}-y_{i}\right)}^{2}}.$$

In Equation (2), $\left(x_{i},y_{i}\right)$ is the node *i* coordinate, and $\left(x_{j},y_{j}\right)$ is the node *j* coordinate.

(3) $$C\left(A\right)=\sum_{k=1}^{n=|A|}\sqrt{{\left(x_{j}-x_{i}\right)}^{2}+{\left(y_{j}-y_{i}\right)}^{2}}.$$

In Equation (3), $\left(x_{i},y_{i}\right)$ and $\left(x_{j},y_{j}\right)$ are the coordinates of the nodes in the set *A*.

In this paper, the problem of improving the efficiency of the sensor submodularity and reducing the communication cost (C) is transformed into a combination optimization problem. The objective function is as follows.

(4) $$\max_{A\subseteq V}F\left(A\right),s.t.C\left(A\right)\leq B.$$

For the communication cost budget B>0, Equation (4) aims to find the solution set with the maximum mutual information within a low communication cost.

### 2.2. IHACA-COpSPIEL Deployment Method

Krause et al. proposed the pSPIEL algorithm, which is an improved greedy algorithm, but with both a larger number of sensors and a longer communication distance. The ant colony algorithm is easy to combine with other methods and performs well in path optimization. Therefore, this paper combines the improved ant colony algorithm with the chaotic operator improved pSPIEL algorithm, and proposes the IHACA-COpSPIEL.

#### 2.2.1. Chaos Optimized pSPIEL Algorithm

The standard pSPIEL algorithm applies non-decreasability, submodularity, and locality to solving the problem of sensor node deployment. Compared with the greedy algorithm, pSPIEL can optimize the sensor layout and reduce the communication cost, but with a large number of sensors and slightly higher communication costs. The ergodicity of chaotic motion can effectively traverse each state within a specified range. Therefore, this paper introduces a chaotic operator to traverse all cluster numbers to determine the optimal cluster number. We propose a chaotic optimized pSPIEL algorithm (COpSPIEL). The basic idea of the chaotic locality parameter *r* adjustment strategy is to use a chaos generator to generate a set of chaotic variables and then use the carrier transform method to map to the locality parameters, and map it to the value range of the locality parameters. Logistic mapping is a typical chaotic system.

(5) $$z_{i+1}=\mu z_{i}\left(1-z_{i}\right),i=0,1,2,\ldots ,z_{i}\in \left(0,1\right).$$

In Equation (5), μ is the control parameter. When μ=4, the system is completely chaotic.

The search $r_{i}$ is mapped to the domain (0,1) of the logistic equation by Equation (6).

(6) $$z_{i}=\left(r_{i}-r_{\min}\right)/\left(r_{\max}-r_{\min}\right),r_{i}\in \left(r_{\min},r_{\max}\right).$$

It iteratively generates a chaotic sequence by Logistic equation:

(7) $$z^{m}\left(m=1,2,3,\ldots \right).$$

The generated chaotic sequence is inversely mapped by Equation (8):

(8) $$r_{i}^{m}=r_{\min}+\left(r_{\max}-r_{\min}\right)z^{m}.$$

This returns to the original solution space and produces a solvable chaotic sequence containing chaotic variables.

(9) $$r_{i}^{m}=\left(r_{1}^{m},r_{2}^{m},\ldots ,r_{i}^{m}\right)$$

The locality parameter *r* optimizes the search space in this sequence.

#### 2.2.2. Improved Heuristic Ant Colony Algorithm

The ant colony algorithm was inspired by the research on real ant colony behavior, and has been applied to the optimization of communication networks and others. The essence of the ant colony algorithm is to use pheromone as a medium for ants in an ant colony to communicate. In the sensor layout, the ants must be moved toward the sensor node with a large submodular gain. The traditional ACA algorithm has the problem of blind search and it is easy to fall into local optimal solutions. To improve the heuristic function and pheromone, a new mechanism, improved heuristic ant colony algorithm, IHACA is proposed.

The heuristic function of the traditional ant colony algorithm takes no consideration of the distance relationship between the next node *j* and the adjacent cluster head, and the search is blind. Therefore, this paper adds the Euclidean distance between the next node j and the cluster head of the adjacent cluster. The improved heuristic function is as follows.

(10) $$\eta_{ij}=\frac{1}{wdist\left(i,j\right)+\left(1-w\right)dist\left(j,g_{i1}\right)},w\in \left(0,1\right).$$

where $g_{i1}$ is the first node of cluster *i* and *w* is the weight.

In order to avoid premature, stagnation or local optimization problems due to excessive pheromone concentration [27], this paper introduces a local and global pheromone update mechanism. Local update of pheromone helps ants to select unselected points, and a full update of pheromone helps to enhance the global search ability of the algorithm.

Each ant moves from node *i* to node *j*, and needs to update the pheromone on the path (i,j) that it just walked.

(11) $$\tau_{ij}\left(n+1\right)=\xi \tau_{ij}\left(n\right)+\varepsilon \tau_{0}.$$

In Equation (11), *n* is the number of iterations, ξ is a local pheromone evaporation coefficient, $\tau_{0}$ is a pheromone under initial conditions, and ε is a constant.

When all the ants complete this iteration, we select the shortest path and the longest path in this iteration to globally update the pheromone on the path.

(12) $$\tau_{ij}\left(n+1\right)=\left(1-\rho \right)\tau_{ij}\left(n\right)+\rho \sum_{k=1}^{m}\Delta \tau_{ij}^{k}.$$

(13) $$\Delta \tau_{ij}^{k}=\left\{\begin{array}{c} \frac{Q}{L_{best}},L\in L_{best}\\ -\frac{Q}{L_{worst}},L\in L_{worst}\\ 0,other \end{array}\right.$$

In Equations (12) and (13), *m* is the number of ants, ρ is the evaporation coefficient of the global information system, $\tau_{ij}^{k}$ is the pheromone left by ant *k* on (i,j), *Q* is the pheromone quality coefficient, $L_{best}$ is the shortest path, and $L_{worst}$ is the longest path.

#### 2.2.3. IHACA-COpSPIEL Algorithm

(1) Clustering 

Using the local parameter αr chaotic sequence pair of Equation (9) randomly divides the position set *V* into small clusters of diameter αr, where α∈(0,1]. The nodes near their cluster boundaries are stripped, so the clusters are well separated. The locality of F makes the clusters almost independent and provides a wealth of information [13].

(2) Establishing module approximation 

In the *i*th cluster ($C_{i}$), a greedy algorithm is used to obtain the ranks of $g_{i,1},g_{i,2},\ldots ,g_{i,n_{i}}$ on the *i*th cluster’s nodes ($n_{i}$), and the nodes are connected in this order to form a chain of clusters. A module approximation graph $G^{\prime }$ is created from *G* through these chains. A modular directional arithmetic algorithm is used on $G^{\prime }$ to solve the corresponding objective function, the selected path in $G^{\prime }$ is extended according to the corresponding shortest path in *G*, and the solution set $A^{\prime }$ is output.

(3) Select the next position 

The initial node of $A^{\prime }$ is used as the initial value of the Improved Heuristic Ant Colony Algorithm (IHACA). The IHACA algorithm selects the next position from the first node according to Equation (14), and adds the selected position to the taboo table or $tabu_{k}$ of ant *k*. $\eta_{ij}^{\beta }$ is calculated by Equation (10), and $\tau_{ij}$ is calculated by Equation (15).

(14) $$P_{ij}^{k}=\frac{\tau_{ij}^{\alpha }\eta_{ij}^{\beta }}{\sum_{j\in A}\tau_{ij}^{\alpha }\eta_{ij}^{\beta }}$$

(15) $$\tau_{ij}=F\left(C_{i}\cup k_{j}\right)-F\left(C_{i}\right).$$

In Equation (14), α is the weight of the path, β is the weight of the heuristic information, and $\tau_{ij}$ represents the pheromone intensity of the path from the cluster $C_{i}$ to the $k_{j}$ sensor.

(4) Pheromone update 

After the next position is determined, the pheromone traversed by the ant (i,j) is updated according to Equation (11). When all ants reach the endpoint, the global pheromone is updated according to Equation (12), and the tabu list is cleared.

The pseudo-code of the IHACA-COpSPIEL algorithm is as follows. Line 2 calls clustering with complexity $O(m_{c\max})$. Lines 3–6 form a chain with complexity O(NlogN). Line 7 calls the block-oriented algorithm with complexity O(nlogN) where *n* is the number of connectable edges of graph nodes (n≤N). Lines 8–10 select nodes for $A^{{}^{\prime \prime }}$ of the greedy algorithm with complexity $O(log(N-n_{i}))$. Lines 13–17 reach the given maximum mutual information with complexity $O(kN^{2})$ where *k* is iteration times. Line 22 calls updating global pheromone with complexity $O(N^{2})$. The computational complexity of Algorithm 1 is $O(kN^{2})$ approximately.

**Algorithm 1** Improved Heuristic Ant Colony Algorithm-Chaos Optimization of Padded Sensor Placements at Informative and cost-Effective Locations (IHACA-COpSPIEL).**Input:** Position set *V* and covariance matrix**Output:** Solution set *A*1: Initialize parameters: α, β, *w*, ξ, $\tau_{0}$, ε, $n_{\max}$2: Divide V into $m_{cmax}$ clusters $\left\{C_{i}|i\in \left[1,m_{c\max}\right]\right\}$3: for each cluster $C_{i}$
do4:  Sort position points in $C_{i}$ by greedy algorithm and then get the ranks of $g_{i,1},g_{i,2},\ldots ,g_{i,n_{i}}$5:  Connect $g_{i,1},g_{i,2},\ldots ,g_{i,n_{i}}$ to form a chain which is then included into ${G_{i}}^{{}^{\prime }}$6: end7: Uses $G^{{}^{\prime }}$ as input of block-oriented algorithm to solve F(A) and then get the solution $A^{{}^{\prime \prime }}$, where $G^{{}^{\prime }}$ = $\left\{{G_{i}}^{{}^{\prime }}|i\in \left[1,m_{c\max}\right]\right\}$8: while a given maximum mutural information in $A^{{}^{\prime \prime }}$ is not reached do9:   Select nodes for $A^{{}^{\prime \prime }}$ with greedy algorithm10: end11: $A^{{}^{\prime }}$=$A^{{}^{\prime \prime }}$12: for n=1:$n_{max}$
do13:   while a given maximum mutural information in ${A_{n}}^{{}^{\prime }}$ is not reached do14:   Select IHACA initial points in ${A_{n}}^{{}^{\prime }}$ from head nodes in $A^{{}^{\prime }}$15:   Select next point with Equation (14)16:   Update local pheromone $\tau_{ij}\left(n\right)$ with Equation (11)17:  end18:  if
$C({A_{n}}^{{}^{\prime }})\leq B$
then19:   *A*=${A_{n}}^{{}^{\prime }}$ and output *A*20:   return21:  end22:  Update global pheromone $\tau_{ij}\left(n\right)$ with Equation (12)23: end24: *A*=${A_{n}}^{{}^{\prime }}$ and output *A*25: return

## 3. Routing Protocols for Wireless Sensor Networks

### 3.1. Communication Model

The energy consumption of data sent by sensor nodes is shown in Equation (16).

(16) $$E_{TX}\left(k,d\right)=E_{elec}\left(k\right)+E_{amp}\left(k,d\right)=\left\{\begin{array}{c} kE_{elec}+kE_{fs}d^{2},d<d_{0}\\ kE_{elec}+kE_{mp}d^{4},d\geq d_{0} \end{array}\right.,$$

where *k* is the number of bits of transmitted data, *d* is the transmission distance, $E_{elec}\left(k\right)$ is the energy consumption of the transmitting circuit to send *k* bit data, and $E_{amp}\left(k,d\right)$ is the transmission power amplifier transmitting *k* bit data when the transmission distance is *d*. $E_{elec}$ is the unit energy consumption of the transmitting or receiving circuit, and $d_{0}$ is the threshold. $E_{fs}$ is the energy consumption parameter of the transmission power amplifier under the free space channel model and $E_{mp}$ is the energy consumption parameter of the transmission power amplifier under the multipath fading channel model.

The calculation of the energy consumption of the receiving circuit to receive *k* bit data is shown in Equation (17).

(17) $$E_{RX}\left(k\right)=E_{TX-elec}\left(k\right)=kE_{elec}.$$

### 3.2. Optimal Clustering

The number of cluster heads has a great impact on network performance. According to [14], the optimal number of cluster heads is shown in Equation (18).

(18) $$k_{opt}=\frac{\sqrt{N_{A}\cdot E_{fs}}}{\sqrt{2\pi \cdot E_{mp}}}\frac{M}{d_{{}_{toBS}}^{2}}.$$

In Equation (19), $N_{A}$ is the number of nodes in set *A*, *M* is the area side length, and $d_{{}_{toBS}}$ is the distance from the node to the base station.

The probability of a node being elected as a cluster head is shown in Equation (19).

(19) $$p=\frac{k_{opt}}{N_{rounds}}.$$

### 3.3. Fitness Function

The fitness values are based on parameters used to achieve the best solution. It considers intra-cluster compactness, inter-cluster separation and total energy consumption.

Tightness refers to the internal distance, that is, the distance between the nodes in the cluster and the cluster head (CH).

(20) $$C=\sum_{i=1}^{CHs}\sum_{\forall n\in C_{i}}d(n,CH_{i}).$$

Separability refers to the distance between clusters, that is, the minimum distance between cluster heads.

(21) $$S=\min_{\forall C_{i},C_{j},C_{i}\neq C_{j}}\left(d\left(CH_{i},CH_{j}\right)\right).$$

The total energy consumption refers to both the cluster head communication energy consumption $E_{CH}$ and ordinary node communication energy consumption $E_{NN}$, of which the energy consumption of the cluster head includes the energy consumption $E_{RN}$ required to receive data sent by the nodes in the cluster, the energy consumption $E_{HD}$ required to collect data for fusion, and the energy consumption $E_{TB}$ required to send data to the base station. The energy consumption of ordinary nodes includes the energy consumption $E_{TH}$ required to send data to the cluster head. Assume that the total number of nodes is $N_{A}$, the number of cluster heads is *m*, and the ordinary nodes in each cluster are $n_{1}$, $n_{2}$,..., $n_{m}$.

(22) $$E_{CH}=E_{RN}+E_{HD}+E_{TB}=kE_{elec}n_{i}+kE_{DA}\left(n_{i}+1\right)+\left(kE_{elec}+kE_{mp}l_{HB}^{4}\right).$$

In Equation (16), $E_{DA}$ is the energy consumed by unit bit data fusion, and $l_{HB}$ is the distance between the cluster head and the base station.

(23) $$E_{NN}=E_{TH}=kE_{elec}+kE_{fs}l_{NH}^{2}.$$

In Equation (17), $l_{NH}$ is the distance between the nodes in the cluster and the cluster head.

(24) $$E_{total}=\sum_{i=1}^{m}E_{CH_{i}}+\sum_{j=1}^{N}E_{NN_{j}}.$$

In Equation (18), the closer the distance between the cluster nodes and the cluster head in a cluster, the better. The greater the separation between cluster heads, the better the total energy consumption. The fitness function is as follows.

(25) $$F=w_{1}C+\frac{w_{2}}{S}+w_{3}E_{total},w_{1},w_{2},w_{3}\in \left(0,1\right).$$

In Equation (19), $w_{1}$+$w_{2}$+$w_{3}$=1.

### 3.4. Routing Protocol Based on BBO Algorithm

BBO algorithm is an information intelligence heuristic algorithm first proposed by Dan Simon in 2008. The habitats of biological populations have their corresponding Habitat Suitability Index (HSI), which is used to describe the quality of the habitat environment, and the factors that affect the fitness index are called Suitable Index Variables (SIVs). The BBO algorithm has the advantages of simple operation, fast convergence, and fewer parameters [28]. The standard BBO algorithm uses a simple linear migration model, but in the real biogeographic environment, species migration often occurs randomly and does not follow this rule. Complex and natural migration models are much better than simple migration models [23,29]. In this paper, a cosine migration model is used. When the number of species in the habitat is either large or small, the change in the immigration rate λ and the emigration rate μ is relatively stable. When the number of species in the habitat is neither large nor small, the immigration rate λ and the emigration rate μ changes quickly. The expression of the cosine migration model is shown in Equations (26) and (27).

(26) $$\lambda_{k}=\frac{I}{2}\left(\cos\left(\frac{k\pi }{n}\right)+1\right)$$

(27) $$\mu_{k}=\frac{E}{2}\left(-\cos\left(\frac{k\pi }{n}\right)+1\right).$$

In Equations (26) and (27), *I* is the maximum value of the immigration rate, *E* is the maximum value of the emigration rate, *k* is population number and *n* is the maximum population number.

The mutation operator provides a certain global search capability for the algorithm through the mutation of the habitat’s own information.

(28) $$m_{i}=m_{\max}\left(1-\frac{p_{s}}{p_{\max}}\right).$$

In Equation (28), $m_{max}$ is the maximum mutation rate, $p_{s}$ is the probability that habitat *i* has *s* species, and $p_{max}$ = $max(p_{s})$.

The steps of optimizing wireless sensor network routing protocol based on the BBO algorithm are as follows. Lines 3–7 select CH with complexity $O(n^{2})$. Lines 9–31 reach the minimum fitness value with complexity $O(qn^{2})$ where *q* is the number of iterations. Lines 10–20 calculate the migration rate with complexity $O(n^{2})$. Lines 21–28 calculate the mutation rate with complexity O(n) and lines 32–35 calculate with complexity $O(m_{2})$ where $m_{2}$ is the number of nodes ($m_{2}\geq n$). The computational complexity of Algorithms 2 is $O(qn^{2})$ per round approximately.

**Algorithm 2** Biogeography-Based Optimization (BBO)-based routing protocol process.**Input:** node coordinates, energy model**Output:** residual energy per round, number of dead nodes, number of surviving nodes1: Initialize parameters: number of habitats *n*, maximum emigration rate *E*, maximum immigration rate *I*, probability of species number for each habitat $p_{s}$, maximum number of species $p_{max}$, maximum number of rounds $round_{max}$2: for
*l* = 1: $round_{max}$
do3:  for
*j* = 1: *n*
do4:   Select CH according to Equation (19)5:   Initialize population randomly6:   Calculate the fitness value of habitat *j* according to Equation (25)7:  end8:  Keep habitat with the smallest fitness values as elite habitat9:  while habitat does not reach minimum fitness value do10:   for k = 1: *n*
do11:    Calculate the migration rate $\lambda_{k}$ according to Equation (26)12:    If
$\lambda_{k}$ is greater than a uniformly distributed pseudo random number in [0,1] then13:     for t = 1: *n*
do14:      Calculate the migration rate $\mu_{t}$ according to Equation (27)15:      If
$\mu_{t}$ is greater than a uniformly distributed pseudo random number in [0,1] then16:       The roulette selection method is used to select the population to move out of the habitat *t*       and move into the habitat *k*17:      end18:     end19:    end20:   end21:   for
*i* = 1: *n*
do22:    If Habitat *i* is not an elite habitat then23:     Calculate the mutation rate $m_{i}$ according to Equation (28)24:     If
$m_{i}$ is greater than a uniformly distributed pseudo random number in [0,1] then25:      Select population mutations in habitat *i* randomly26:     end27:    end28:   end29:   Calculate fitness value30:   Replace the worst habitats with elite habitats31:  end32:  Calculate the shortest distance from ordinary nodes to CH33:  Calculate the energy consumed by ordinary nodes to CH to transmit and receive data packets34:  Calculate the energy consumed by CH to sink nodes to transmit and receive data packets35:  Calculate the remaining energy, dead nodes, and surviving nodes of the sensor network36:  If All network nodes are dead then37:   return38:  end39: end40: return

## 4. Experimental Verification

### 4.1. Parameter Settings

One of the application backgrounds of this research is for forest and grassland fire risk monitoring and early warning. The precision and accuracy of forest and grassland fire risk monitoring depend on the multi-source and space-time data which are collected under the high-resolution sensor layout. How to deploy as few sensors as possible to monitor a large area of forests and grasslands and how to measure such multi-source parameters related to fire risk warning as atmospheric temperature, light, soil temperature and humidity, wind speed and rainfall are the application problems that need to be solved. To meet the application requirements, this paper takes the distance between and among the wireless sensor nodes into consideration. The service time and life of the whole network are extended to the maximum extent under such constraints as communication energy consumption and node distances. In order to verify the comprehensive performance of the algorithm proposed in this paper, we conducted simulation experiments and comparisons among the four algorithms, i.e., the greedy algorithm, the pSPIEL algorithm, the ant colony algorithm, and the improved heuristic ant colony algorithm. The BBO-based routing protocol is used for data transmission. In this paper, the forest environment monitoring area is separated into |V|=N=86 locations, and a subset is selected to deploy sensors. The experimental parameter settings are shown in Table 1. We explain the experimental parameter settings as follows.

In Algorithm 1, α is a parameter in Equation (14). If α is large, it would make the ant search according to the pheromone and fall into the local minimum easily, whereas if α is small and its value is 0.1, it would increase the randomness of the search. For the same reason, β in Equation (14), ξ in Equation (11), and ρ in Equation (12) are all 0.1. *w* is the weight parameter of the heuristic function in Equation (10). To achieve equilibrium of the distance effects from node *j* to node *i* and from node *j* to head of adjacent cluster on the heuristic function, *w* takes the value of 0.5. *Q* is a parameter in Equation (13) and its value is 1 in order to strengthen the positive feedback mechanism of the algorithm. $\tau_{0}$ is a parameter in Equation (11) and it takes a small value of 0.0003 to increase the probability for ant to choose an optimal path. ε is a parameter in Equation (11), which takes constant value 1. $n_{max}$ is greater than *N*.

In Algorithm 2, $p_{s}$ is a parameter in Equation (28). The smaller the $p_{s}$ value is, the more it is prone to mutation. Hence the $p_{s}$ value is 0.1. $p_{max}$ is a parameter in Equation (28). It is the maximum value of $p_{s}$ and it takes a value of 1. *I* is a parameter in Equation (26) and *E* is a parameter in Equation (27). In order to make both the immigration rate and the emigration rate take value at [0,1], both *I* and *E* take value 1. $E_{0}$, $E_{elec}$, $E_{mp}$ and $E_{fs}$ take the commonly used default values. The value $round_{max}$ depends on the lifetime of all nodes of the network.

### 4.2. Results and Analyses

(1) Comparison of communication cost and the number of sensors 

**Figure 1:**
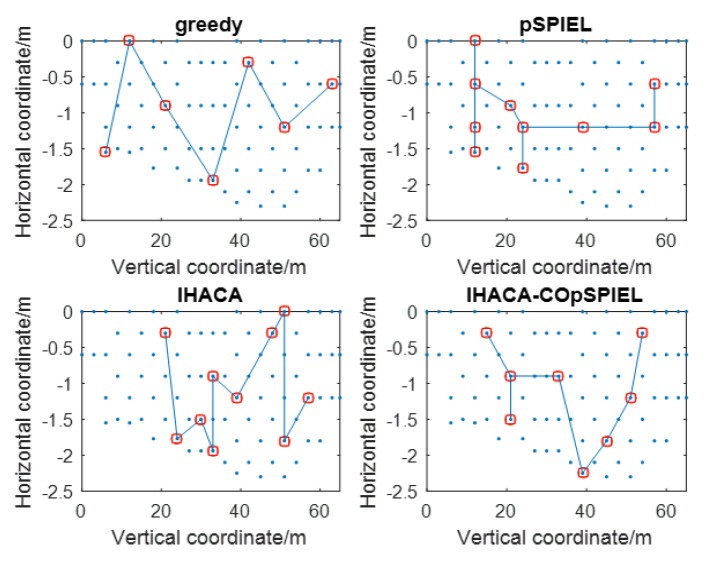
Sensor deployments of the four algorithms.

Table 2 is a set of experimental data of the simulation. Table 2 and Table 3 compare the greedy algorithm, the pSPIEL algorithm, and the IHACA algorithm, respectively. The algorithm proposed in this paper not only meets the same deployment requirements but also achieves the best results, that is, the lowest communication cost and the number of sensors. With the same amount of mutual information and compared with the greedy algorithm, the pSPIEL algorithm, and the IHACA algorithm, the communication cost of the IHACA-COpSPIEL algorithm is also the lowest. When mutual information is 0.16, sensor deployment has a high-cost performance, and the communication cost of the IHACA-COpSPIEL algorithm is 38.42% lower than that of the greedy algorithm, 24.19% lower than that of the pSPIEL algorithm, and 8.31% lower than that of the IHACA algorithm. The sensor deployments of the four algorithms are shown in Figure 1, where the blue dots indicate the possibly-deployed points and the red squares indicate the selected points for deployment.

The *r* value of the pSPIEL algorithm is randomly selected and the number of clusters is also random. Thus the number of clusters affects the selection of nodes and it is difficult to obtain the optimal number of clusters. Therefore the communication cost is high. The IHACA-COpSPIEL algorithm adds a chaotic operator, which can traverse the local parameter *r* value to obtain the number of clusters under different *r* values. The deployed nodes are selected within the optimal number of clusters. The heuristic function concerns the distance between the next node and the adjacent cluster head to minimize the communication distance between sensors. The first node of the solution set of the COpSPIEL algorithm is used as the first node of the IHACA. The node with maximum mutual information is selected as the deployment point. This can reduce the number of sensors and reduce the total communication cost, so it has better performance than any other algorithm.

The objective function of sensor deployment is submodular. When mutual information was 0.14 and the number of sensors was small, or when mutual information was 0.20 and the number of sensors was large, a sensor is added each time and the communication cost was the lowest compared with the greedy algorithm, the pSPIEL algorithm, and the IHACA algorithm. The more the amount of mutual information increases as the number of sensors increases, the better the sensor deployment effect. When the number of sensors is small, the increment of submodular benefit is large by adding a new sensor each time. As the number of sensors increases, the increment of submodular benefit starts to decrease whenever a new sensor is added.

**Figure 2:**
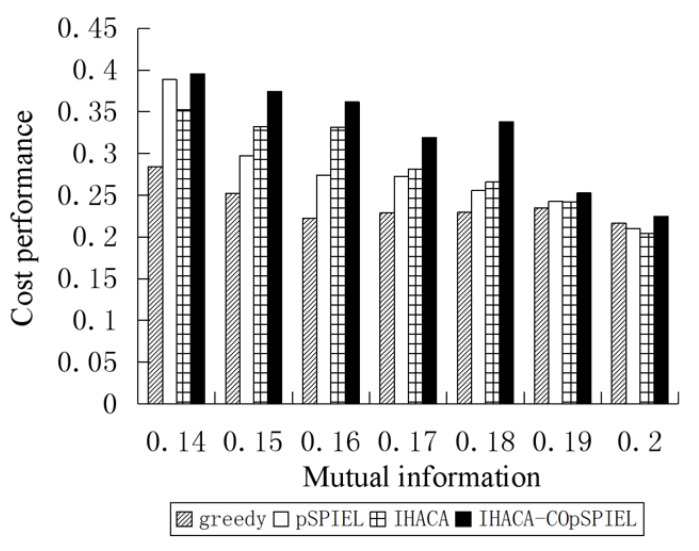
Cost performance and mutual information of the four algorithms.

Figure 2 shows that under the mutual information of 0.14–0.20, the ratio of the IHACA-COpSPIEL algorithm is higher than that of the greedy algorithm, the pSPIEL algorithm, and the IHACA algorithm. Therefore, IHACA-CpSPIEL achieves the best cost-benefit ratio.

(2) Comparison of the lifecycle and average energy 

**Figure 3:**
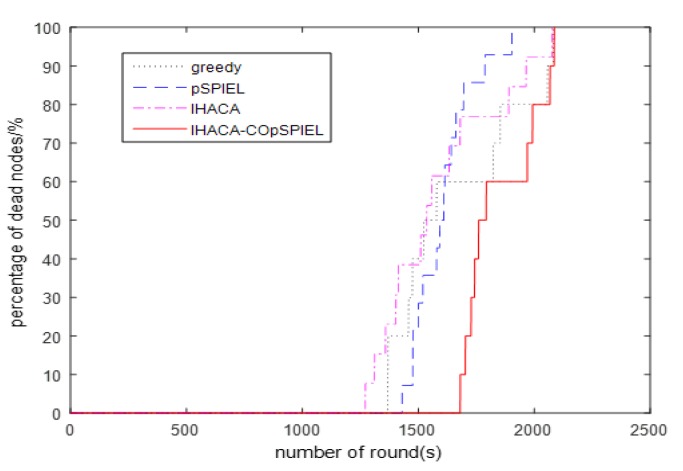
Comparison of the percentage of dead nodes in the four algorithms.

**Figure 4:**
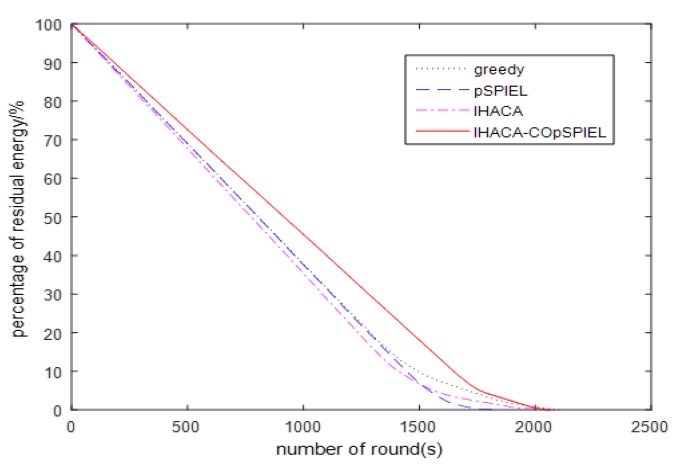
Percentage of remaining energy in the four algorithms.

The life cycle refers to the time from which the wireless sensor starts to work until the death of the first node. Figure 3 shows the comparison results of the life cycle of the sensor nodes selected by the routing protocol based on the BBO algorithm for the greedy algorithm, the pSPIEL algorithm, the IHACA algorithm, and the IHACA-COpSPIEL algorithm proposed in this paper. It shows from Figure 3 that the first dead node under the greedy algorithm appears in round 1368, the first dead node in the pSPIEL algorithm appears in round 1430, the first dead node in the IHACA algorithm appears in round 1272, and the first dead node of the IHACA-COpSPIEL algorithm proposed in this paper appears in round 1681, which indicates that the wireless sensors deployed in the IHACA-COpSPIEL algorithm have a longer life cycle. The reason for this is that the communication distance of the wireless sensors deployed by the IHACA-COpSPIEL algorithm is the shortest, which reduces the energy consumption of transmission. Figure 4 shows a comparison of the remaining energy percentages of data transmission after the sensors are deployed in each of the four algorithms. The IHACA-COpSPIEL algorithm has a higher percentage of remaining energy per round than the greedy, pSPIEL, and IHACA algorithms. Therefore, the overall energy consumption of the proposed algorithm is lower than any of the other three algorithms.

**Figure 5:**
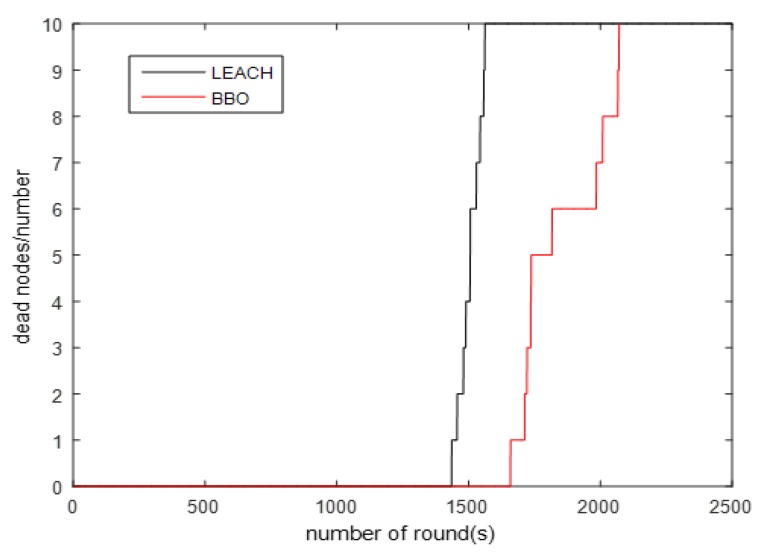
Comparison of dead nodes under two protocols.

**Figure 6:**
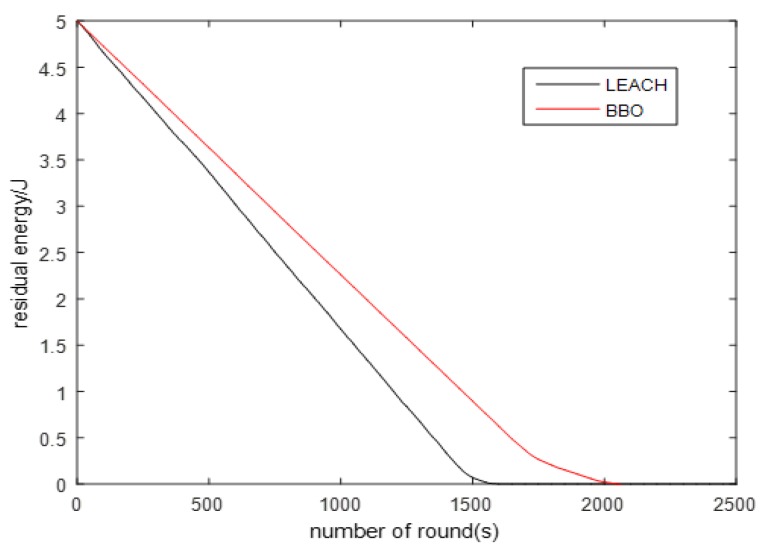
Comparison of residual energy under two protocols.

As the number of simulation rounds increases, Figure 5 and Figure 6 show both the deaths of nodes by using the LEACH routing protocol and the BBO routing protocol, respectively, and the network energy consumption results by using the LEACH routing protocol and the BBO routing protocol, respectively, to deploy sensors in the IHACA-COpSPIEL algorithm. As is seen from Figure 5, under the LEACH protocol, the first node died in round 1435 and the last node died in round 1584, and under the BBO routing protocol, the first node died in round 1659 and all nodes died in round 2071. The network survival time is longer than the former. As is seen from Figure 6 that the remaining energy under the routing protocol based on the BBO algorithm has always been more than that under the LEACH protocol. This is because the routing protocol based on the BBO algorithm fully takes the cluster into account. The distance between the nodes and the cluster heads, the distance between the cluster heads and the cluster heads, and the total energy consumption effectively balances the network load. Hence the life of the entire wireless sensor network is significantly extended.

## 5. Conclusions

In order to reduce costs and save energy, this paper proposes a large-scale sensor deployment method called the IHACA-COpSPIEL algorithm and a routing protocol based on the BBO algorithm. Mutual information is introduced to describe the correlation between observed points and unobserved points, a mathematical model with submodularity is established, and the edges of graph theory are used to represent communication costs. The pSPIEL algorithm with enhanced optimization ability by a chaos operator and the ant colony algorithm with improved heuristic function and pheromone update mechanism are used to find the optimal path. What has been studied can further solve the sensor deployment problem under the constraint of communication cost. Finally, the BBO algorithm-based routing protocol transmits data to the deployed sensors. The computational complexity of the IHACA-COpSPIEL is $O(kN^{2})$, and the computational complexity of the routing protocol based on the BBO algorithm is $O(qn^{2})$. The experiments show that the deployment algorithm proposed in this paper has better sensor deployment capabilities. This deployment algorithm reduces the communication cost by 38.42% compared with the greedy algorithm. It also reduces the number of sensors and has a longer life cycle. Compared with the LEACH protocol, the BBO algorithm-based routing protocol has lower energy consumption and longer network life.

In the future, we intend to use a discrete event simulator (DES) such as NS-3 to further combine practical application scenarios to improve the effectiveness of the algorithm. Our vision for future work is as follows.

We will complete the IHACA-COpSPIEL protocol design in the NS-3. We will refer to the RFC document of Multi-Protocol Label Switching protocol, and elaborate on the design and implementation of each basic component of IHACA-COpSPIEL, including the forwarding equivalence class (FEC), next hop label forwarding entry (NHLFE), FEC to NHLFE mapping (FTN), etc. By statically configuring the label forwarding table, the communication between private networks through the backbone network by IHACA-COpSPIEL forwarding will be realized.

## Figures and Tables

**Table 1:** Parameter settings.

Parameter	Description	Value
*N*	Total of location number for possibly-deployed sensors	86
α	Path weight	0.1
β	Heuristic information weight	0.1
ξ	Local pheromone evaporation coefficient	0.1
ρ	Global information system evaporation coefficient	0.1
*w*	Weight	0.5
*Q*	Pheromone quality coefficient	1
$\tau_{0}$	Pheromone under initial conditions	0.0003
ε	Constant	1
$p_{s}$	Probability that habitat *i* has species *s*	0.1
$p_{max}$	Maximum of $p_{s}$	1
*I*	Maximum immigration rate	1
*E*	Maximum emigration rate	1
$E_{0}$	Sensor node’s initial energy	0.5 J
$E_{elec}$	Energy consumption per bit for transmitting data	50 nJ/bit
$E_{mp}$	Energy consumption of multipath model transmitter	0.013 pJ/bit/m${}^{4}$
$E_{fs}$	Energy consumption of free space model transmitter	10 pJ/bit/m${}^{2}$
$round_{max}$	Maximum round number	2500
$n_{max}$	Maximum iteration number	100

**Table 2:** Comparison of communication cost for the four algorithms.

Mutual Information	Communication Cost			
	Greedy	pSPIEL	IHACA	IHACA-COpSPIEL
0.14	49.22	36.01	39.7	35.42
0.15	59.47	50.4	45.16	40.06
0.16	71.83	58.34	48.24	44.23
0.17	74.31	62.44	60.36	53.26
0.18	78.48	70.43	67.75	64.11
0.19	80.82	78.28	78.41	75.22
0.20	98.57	95.21	97.76	89.05

**Table 3:** Comparison of sensor number for the four algorithms.

Mutual Information	Sensor Number			
	Greedy	pSPLIE	IHACA	IHACA-COpSPIEL
0.14	5	6	7	6
0.15	6	8	8	7
0.16	7	10	10	8
0.17	10	13	12	10
0.18	12	14	13	12
0.19	17	25	25	20
0.20	25	30	30	28

## Abbreviations

The following abbreviations are used in this manuscript:

BBO	Biogeography-Based Optimization
IHACA	Improved Heuristic Ant Colony Algorithm
pSPIEL	padded Sensor Placements at Informative and cost-Effective Locations
IHACA-COpSPIEL	Improved Heuristic Ant Colony Algorithm-Chaos Optimization of padded Sensor
	Placements at Informative and cost-Effective Locations
SIVs	Suitable Index Variables
